# Assessment of the anthelmintic activity of medicinal plant extracts and purified condensed tannins against free-living and parasitic stages of *Oesophagostomum dentatum*

**DOI:** 10.1186/s13071-014-0518-2

**Published:** 2014-11-19

**Authors:** Andrew R Williams, Honorata M Ropiak, Christos Fryganas, Olivier Desrues, Irene Mueller-Harvey, Stig M Thamsborg

**Affiliations:** Department of Veterinary Disease Biology, Faculty of Health and Medical Sciences, University of Copenhagen, Frederiksberg, Denmark; Chemistry and Biochemistry Laboratory, School of Agriculture, Policy and Development, University of Reading, Reading, UK

**Keywords:** *Oesophagostomum dentatum*, Plant extracts, Condensed tannins, Anthelmintic

## Abstract

**Background:**

Plant-derived condensed tannins (CT) show promise as a complementary option to treat gastrointestinal helminth infections, thus reducing reliance on synthetic anthelmintic drugs. Most studies on the anthelmintic effects of CT have been conducted on parasites of ruminant livestock. *Oesophagostomum dentatum* is an economically important parasite of pigs, as well as serving as a useful laboratory model of helminth parasites due to the ability to culture it *in vitro* for long periods through several life-cycle stages. Here, we investigated the anthelmintic effects of CT on multiple life cycle stages of *O. dentatum.*

**Methods:**

Extracts and purified fractions were prepared from five plants containing CT and analysed by HPLC-MS. Anthelmintic activity was assessed at five different stages of the *O. dentatum* life cycle; the development of eggs to infective third-stage larvae (L3), the parasitic L3 stage, the moult from L3 to fourth-stage larvae (L4), the L4 stage and the adult stage.

**Results:**

Free-living larvae of *O. dentatum* were highly susceptible to all five plant extracts. In contrast, only two of the five extracts had activity against L3, as evidenced by migration inhibition assays, whilst three of the five extracts inhibited the moulting of L3 to L4. All five extracts reduced the motility of L4, and the motility of adult worms exposed to a CT-rich extract derived from hazelnut skins was strongly inhibited, with electron microscopy demonstrating direct damage to the worm cuticle and hypodermis. Purified CT fractions retained anthelmintic activity, and depletion of CT from extracts by pre-incubation in polyvinylpolypyrrolidone removed anthelmintic effects, strongly suggesting CT as the active molecules.

**Conclusions:**

These results suggest that CT may have promise as an alternative parasite control option for *O. dentatum* in pigs, particularly against adult stages. Moreover, our results demonstrate a varied susceptibility of different life-cycle stages of the same parasite to CT, which may offer an insight into the anthelmintic mechanisms of these commonly found plant compounds.

## Background

Parasitic worms (helminths) of the gastrointestinal (GI) tract are pathogens of major global importance. Over a billion people, mainly in developing countries, are estimated to be infected with soil-transmitted helminths, whilst helminth infection is also a serious problem in livestock production worldwide, causing significant economic losses and threatening food security [1-3]. Control of helminths relies almost exclusively on a limited number of synthetic anthelmintic drugs. The limitations of this reliance on chemotherapy are the threat of parasites developing resistance to drug treatment (already widespread in some livestock production systems) [4,5] , the cost of drugs for small-scale farmers in developing countries and for some helminths, lack of efficacy of current available drugs [1]. Therefore, novel and complementary helminth control options are urgently needed.

The use of natural plant extracts as de-wormers for humans and livestock has long been practiced, however scientific validation of these practices and identification of active compounds has been lacking [6-8]. Anthelmintic effects of plants are normally ascribed to secondary metabolites such as alkaloids, terpenoids or polyphenols such as proanthocyanidins [9], also known as condensed tannins (CT). Proanthocyanidins are a diverse and widely-occurring group of compounds, and consist of polymers of either catechin and/or epicatechin (termed procyanidins - PC), or of gallocatechin and/or epigallocatechin (termed prodelphinidins - PD), with hetero-polymers being common [10]. They are found in plant material from both tropical and temperate areas, and have been widely investigated for their antioxidant and anti-inflammatory properties [11,12]. It is also apparent that CT can have anthelmintic effects; reduced worm burdens have been reported in rats administered CT in the diet, or in livestock grazing forages containing CT [13,14]. Moreover, direct anthelmintic effects of purified CT have been confirmed in *in vitro* assays against, amongst others, *Haemonchus contortus* [15]*, Ostertagia ostertagi* [16] and *Ascaris suum* [17]. However, much work remains to be done to establish the spectra of activity of CT, i.e. the range of helminth species that are susceptible, and what stages of the life cycle are targeted by these molecules.

*Oesophagostomum dentatum* is a common helminth parasite that resides in the large intestine of pigs, and is of economic importance as it causes significant production losses for farmers [18-20]. Resistance of *O. dentatum* to levamisole, pyrantel and possibly ivermectin has been reported in Europe [21,22]. Moreover, *O. dentatum* also serves as a good model parasite due to its amenability to culture in the laboratory – several different life-cycle stages can be easily maintained and manipulated, and hence this worm is being increasingly used as a model parasite for biological investigations [23]. In the present study we investigated 1) whether CT have direct anthelmintic activity against *O. dentatum in vitro*, and 2) which stages of the life cycle of this parasite were specifically targeted by CT.

## Methods

### Extraction of plant material and analysis of proanthocyanidins

Five sources of plant material were selected, on the basis of known high concentrations of CT and/or use as traditional medicinal plants. Three traditional medicinal plants were obtained from Flos (Mokrsko, Poland) – these were leaves from blackcurrant (*Ribes nigrum*), flowers from *Tilia* (*Tilia L.*, a mixture of *T. cordata*, *T. platyphyllos* and *T. vulgaris*) and bark from willow (*Salix* spp.). Flowers from white clover (*Trifolium repens*) were obtained from Ziola Kurpi (Jednorozec, Poland). Extraction and analysis were as previously described [17]. Briefly, plant material was extracted with acetone/water (excluding addition of ascorbic acid). To purify CT, extracts were applied to Sephadex LH-20 columns, and eluted with acetone/water (3:7 and 1:1, v/v). Condensed tannin content was quantified by thiolytic degradation [16] and HPLC [17] with the use of dihydroquercetin as external standard. The identification of compounds was confirmed by LC-MS [17]. In addition, skins from hazelnut (*Corylus avellana*), which had previously been shown to contain CT with potent *in vitro* anthelmintic effects against *A. suum* [17], were prepared as previously described.

### Larval development assay with free-living stages

All animal experimentation was conducted under the guidelines and with approval of the Danish Animal Experimentation Inspectorate (Licence number 2010/561-1914). Two 8-week-old pigs were each infected with two doses of 5,000 third-stage larvae (L3) *O. dentatum* by stomach tube, three weeks apart. Upon patency, eggs were isolated from freshly collected faeces by sieving and saturated salt flotation [24].

For the development assay, plant extracts were dissolved in distilled water at appropriate concentrations and added to 96-well plates together with 15% (v/v) of a larval feeding solution (1% yeast extract (Sigma-Aldrich) in Earle’s balanced salt solution), antibiotics (300 U/mL penicillin, 300 μg mL/streptomycin) and antimycotic (10 μg/mL amphotericin B). Negative and positive controls consisted of water and 50 μg/mL levamisole, respectively. One hundred eggs were then added per well and the plates incubated at 25°C in a humidified environment for 7 days. Larvae were then killed by the addition of iodine and the numbers of L3 per well were determined.

### Larval migration assay

Third-stage larvae were produced from the faeces of mono-infected donor pigs by standard copro-culture, collected by Baermann apparatus and stored in water at 10°C. On the day of the assay, L3 were rapidly exsheathed by the addition of 2% sodium hypochlorite (10-15% available chloride, Sigma-Aldrich). L3 were then washed five times in sterile water. To assess the migratory ability of the L3, we performed a migration inhibition assay essentially as described previously [17]. Briefly, larvae were suspended in RPMI 1640 media with HEPES (Gibco) containing L-glutamine (2 mM), 100 U/ mL penicillin and 100 μg/mL streptomycin. One hundred larvae were then added in triplicate to each well of a 48-well plate containing medium with either plant extracts or purified CT fractions, 50 μg/mL ivermectin (positive control) or medium alone (negative control). The larvae were then incubated overnight at 37°C in an atmosphere of 5% CO_2,_ before addition of agar to a final concentration of 0.8%. After a four hour migration period, the numbers of larvae able to migrate out of the setting agar were counted by light microscopy. In some experiments, plant extracts were pre-incubated with polyvinylpolypyrrolidone (PVPP) to selectively deplete CT – this was done by overnight incubation as described previously [17].

### Larval development assay with parasitic stages

Third-stage larvae were exsheathed as above, washed and then 100 larvae per well were seeded in 2 mL of culture medium (LB broth containing 10% heat-inactivated porcine serum, 200 U/mL penicillin, 200 μg/mL streptomycin and 1 μg/mL amphotericin B) in 24-well plates. Acetone/water plant extracts were added at a concentration of 1 mg/mL. Positive control wells received 25 mM of diethylcarbamazine (Sigma-Aldrich) in order to block the development from third-stage to fourth-stage larvae, whilst negative control wells received only culture medium. The larvae were then incubated at 37°C in an atmosphere of 5% CO_2_ for 14 days, with the medium being replenished on days 5 and 10. On day 14 the percentage of L4 in each well was determined.

### Motility assay with fourth-stage larvae

Third-stage larvae were exsheathed as above, washed and then seeded at a concentration of 1000 larvae/mL in tissue culture flasks containing complete LB broth culture medium. Larvae were then incubated at 37°C in an atmosphere of 5% CO_2_ with medium changes every five days. Larvae were cultured for 21–24 days to allow development of large numbers of L4. The L4 were then separated from L3 by repeated sedimentation, washed five times in warm sterile saline, and then suspended in the same medium as used for the L3 migration assay above. Approximately 10 larvae were then added to each well of a 48-well plate containing either acetone/water plant extracts or purified CT fractions, medium only or ivermectin (50 μg/mL). The motility of the larvae was then scored daily for four days. Motility was scored on a 0–5 scale where 0 is no movement and 5 is vigorous movement, as described in more detail by Stepek *et al.* [25].

### Motility assay with adult worms

Donor pigs were infected with *O. dentatum* larvae as described above. Approximately five weeks after the second infective dose, the pigs were killed by captive bolt pistol and exsanguination, and the large intestine was removed. Adult worms were manually plucked from the gut contents with forceps and washed well with warm saline. The worms were then taken to the laboratory and washed repeatedly in the same sterile culture medium as used for the L3 migration assay. Four worms were then seeded in each well of a 24-well plate containing either medium only, ivermectin (50 μg/mL) or hazelnut skin extract. The worms were incubated at 37°C in an atmosphere of 5% CO_2_ for three days, with motility assessed twice daily as described above. After 24 hours, a subset of worms from the negative control wells and from the wells containing the highest concentration of CT were washed, fixed and examined by transmission electron microscopy as previously described [17].

### Statistical analysis

The effect of plant extracts and CT fractions on larval migration and moulting was assessed by two-way ANOVA with Bonferonni post-hoc testing. Graphpad Prism 6 was used for the analyses.

## Results

### Analysis of plant extracts

Acetone/water extraction of the five plant materials yielded extracts with varying yields of CT ranging from 13.1 g CT/100 g extract (white clover flowers) to 73.8 g CT/100 g extract for hazelnut skins (Table 1). Fractionation on Sephadex LH-20 with acetone/water yielded a first fraction (F1) containing from 13.3 to 55.4 g CT/100 g fraction, whilst the second fraction (F2) contained more CT (ranging from 70.3-96.7 g CT/100 g fraction - Table 1).

**Table 1:** **Chemical analysis of plant extracts and derived fractions**

**Sample**	**PAC (g/100 g extract or fraction)**
Hazelnut skin extract	73.8
F1	51.3
F2	70.3
White clover flower extract	13.1
F1	13.3
F2	81.9
Blackcurrant leaves extract	20.2
F1	55.4
F2	76.8
Willow bark extract	16.2
F1	24.2
F2	87.0
*Tilia* flowers extract	21.0
F1	49.7
F2	96.1

‘PAC’ – proanthocyanidins, ‘F1’ – fraction 1, ‘F2’ – fraction 2. Results from the hazelnut skin extract and fractions were also reported previously in [17].

### Tannin-containing extracts inhibit the development of free-living larvae

**Figure 1:**
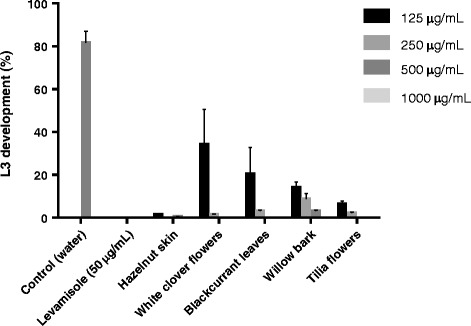
**Inhibition of development of** ***Oesophagostomum dentatum*** **larvae by plant extracts.** Percentage development of *O. dentatum* free living stages, from eggs through L1-L2 to L3, in the presence of water, levamisole or different concentrations from five plant extracts.

Acetone/water extracts were first tested in the widely-used assay of free-living larval development, which measures the ability of newly-hatched larvae from eggs to develop to infective L3 in the presence of a putative anthelmintic agent. In negative control wells, 80% of larvae developed to L3 after 7 days. The addition of 50 μg/mL levamisole resulted in 100% inhibition of egg hatching and therefore subsequent larval development. Egg hatching was not inhibited by the plant extracts, but all five plant extracts strongly inhibited the development of L1 to infective L3, with most larvae dying at the L1 or L2 stage (Figure 1). A dose-dependent relationship was evident for all extracts except for hazelnut skin, where all four tested concentrations (125 – 1000 μg/mL) inhibited development by more than 90% (Figure 1). Overall, these results clearly show that CT-containing plant extracts have anthelmintic activity against the free-living stages of *O. dentatum.*

### Activity of extracts and tannin fractions against parasitic third-stage larvae

**Figure 2:**
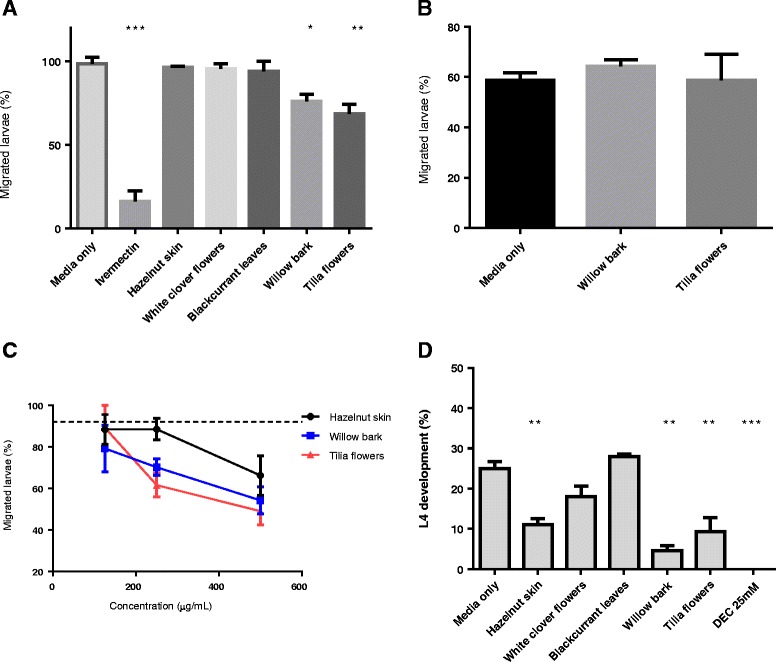
**Activity of plant extracts and purified condensed tannin fractions against** ***Oesophagostomum dentatum*** **third-stage larvae. A** – Percentage *O. dentatum* L3 migration after overnight incubation in media only, ivermectin (50 μg/mL) or plant extracts (1 mg/mL). Results are the mean of two independent experiments, each performed in triplicate. *P < 0.05; **P < 0.01; ***P < 0.001. **B **– Percentage of *O. dentatum* L3 migration after overnight incubation in media only or in plant extracts pre-incubated with PVPP (see materials and methods). Results are mean of two independent experiments, each performed in triplicate. **C** – Percentage of *O. dentatum* L3 migration after overnight incubation in derived F2 ractions from hazelnut skin, willow bark or *Tilia* flowers. Results are mean of two independent experiments, each performed in triplicate. Concentration refers to the amount of CT in each fraction. The dashed line represents the number of migrated larvae after incubation in medium only (negative control). **D** – Percentage development of *O. dentatum* from L3 to L4 after 14 days incubation in medium only, plant extracts (1 mg/mL) or diethylcarbamazine (25 mM). Results are from a single experiment performed in triplicate. **P < 0.01; ***P < 0.001.

Having established that all five extracts had activity against free-living larvae, anthelmintic effects were next assessed against the L3 larval stage that is infective to pigs and develops within the GI tract. First, the migratory ability of exsheathed L3 was quantified after incubation in the extracts. Migration of L3 was reduced (P *<* 0.01) after overnight incubation with extracts of willow bark and *Tilia* flowers at a concentration of 1 mg/mL; in contrast, migration was not significantly reduced after incubation in the other three extracts (Figure 2A). To assess whether CT were the active molecules responsible for the inhibitory activity of willow bark and *Tilia* extracts, the samples were pre-incubated with PVPP, which selectively binds and precipitates CT [10]. Incubation of larvae in these CT-depleted extracts did not affect migratory ability, strongly suggesting CT as the active compounds (Figure 2B).

To further confirm the role of CT in the observed effects, F1 and F2 fractions were isolated from the acetone/water extracts of willow bark and *Tilia* and used in migration inhibition assays. The activity of hazelnut skin fractions, which had previously demonstrated potent anthelmintic effects against *A. suum* [17], were also examined. No anthelmintic activity was evident with the F1 fractions at concentrations up to 500 μg/mL of CT (data not shown). However, F2 fractions inhibited migration in a dose-dependent manner (Figure 2C).

We next investigated whether long-term incubation of L3 in the extracts would reduce subsequent moulting to the L4 larval stage. After 14 days incubation in medium alone, 25% of L3 had moulted to the L4 stage. In contrast, the number of L3 that successfully moulted to L4 was significantly reduced after incubation in extract from hazelnut skins, willow bark or *Tilia* flowers (Figure 2D). No inhibition of moulting was observed after incubation with extracts from white clover flowers or blackcurrant. These data indicate that some CTs are able to interfere with the moulting process of *O. dentatum*.

### Effects of extracts and tannin fractions on motility of fourth-stage larvae

**Figure 3:**
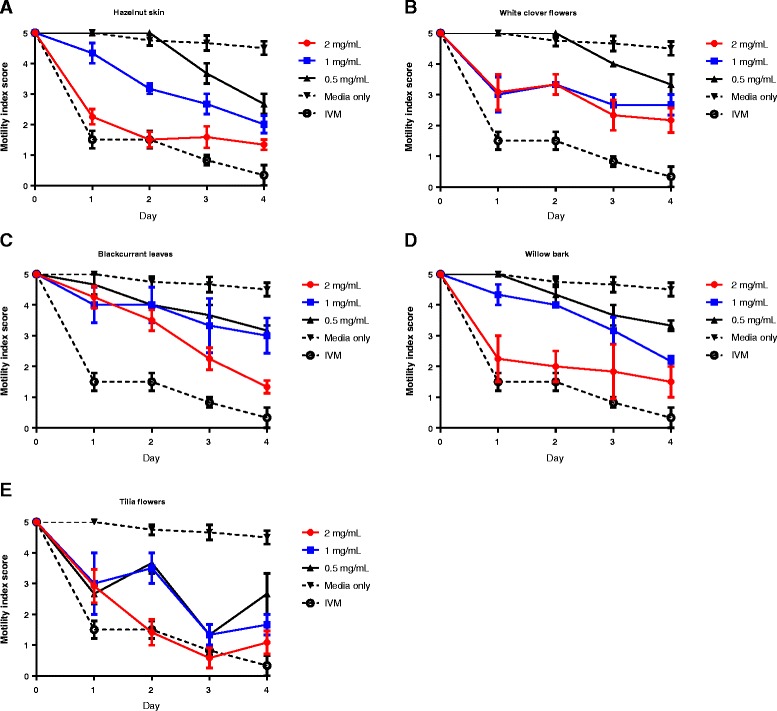
**Effects of plant extracts on motility of** ***Oesophagostomum dentatum*** **fourth-stage larvae.** Inhibition of *O. dentatum* L4 motility by extracts from **A)** Hazelnut skin, **B)** white clover flowers, **C)** blackcurrant leaves, **D)** willow bark and **E)** *Tilia* flowers. Results are mean of two independent experiments, each performed in triplicate. IVM – 50 μg/mL ivermectin.

Anthelmintic effects of the plant extracts and purified CT fractions were further assessed against *in vitro* cultured L4 parasites. After incubation in each of the five plant extracts at concentrations of 2 or 1 mg/mL, a reduction in motility was noted in all parasites after 24 hours (Figure 3). In parasites exposed to extracts from white clover flowers, no further reductions in motility were observed over the next 3 days (Figure 3B), whereas motility continued to decline in worms exposed to the other four extracts, resulting in a near-cessation of movement at the high concentrations of extract by the end of the experiment (Figure 3A, C-E).

**Figure 4:**
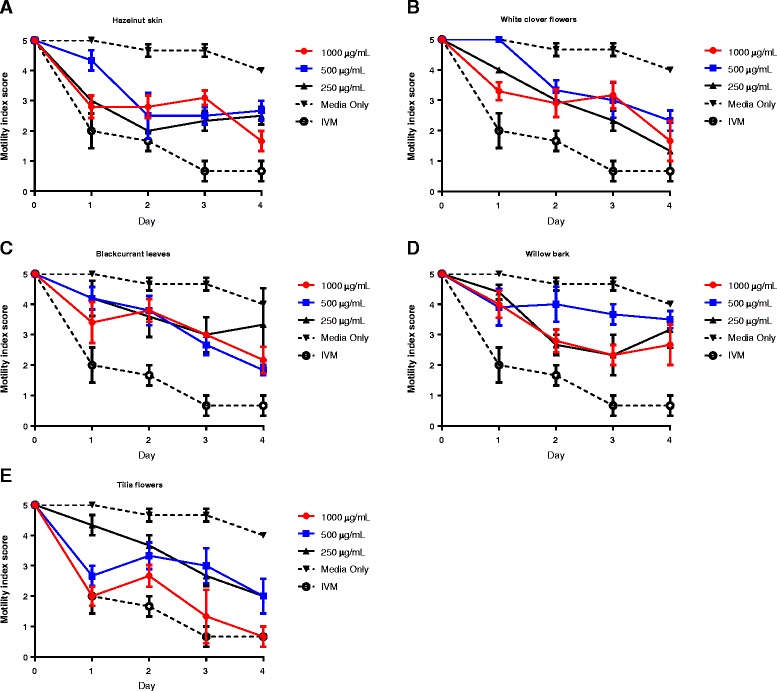
**Effects of purified condensed tannin fractions on motility of** ***Oesophagostomum dentatum*** **fourth-stage larvae.** Inhibition of *O. dentatum* L4 motility by derived F2 fractions from **A)** Hazelnut skin, **B)** white clover flowers, **C)** blackcurrant leaves, **D)** willow bark and **E)** *Tilia* flowers. Results are mean of two independent experiments, each performed in triplicate. Concentrations refer to the amount of CT in each fraction. IVM – 50 μg/mL ivermectin.

To explore the relative contribution of the different CT molecules to the observed effects, isolated F2 fractions from all five extracts were then tested in the motility assay, as anthelmintic activity had been associated with this fraction in the L3 migration assay. The concentrations of CT were normalised between fractions to allow direct comparison between samples. Similar to the results with the acetone/water extracts, incubation in the F2 fractions resulted in reductions of motility after 24 hours of incubation (Figure 4). Motility continued to decline substantially in L4 exposed to fractions of *Tilia* flowers, resulting in near-paralysis by the end of the experiment (Figure 4E). In worms exposed to the other four fractions, further reductions in motility were less apparent, even after four days of incubation; however, motility did not return to pre-incubation levels (Figure 4A-D).

### Effects of hazelnut skin-extract on motility and morphology of adult worms

**Figure 5:**
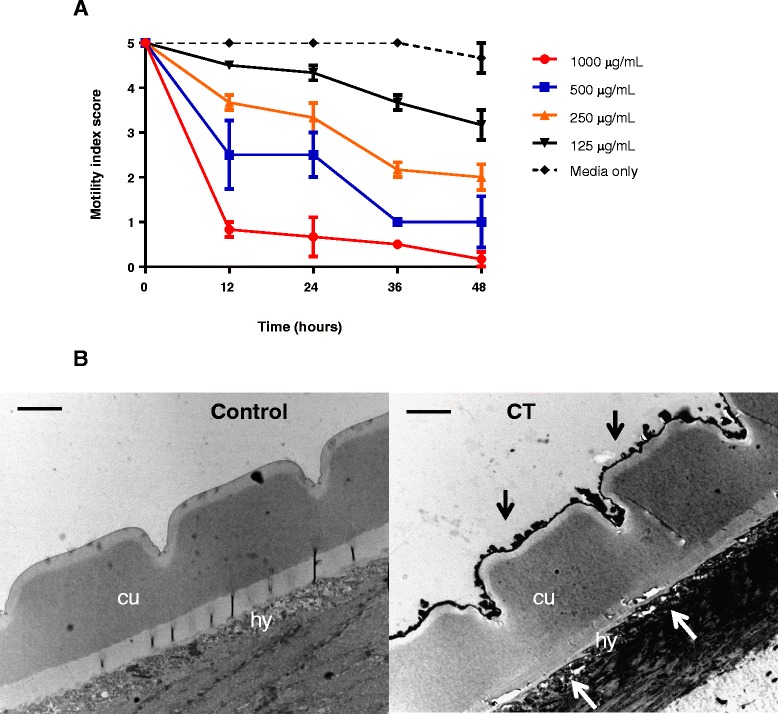
**Effects of condensed tannins on motility and morphology of adult** ***Oesophagostomum dentatum.*** **A** – Inhibition of *O. dentatum* adult motility by hazelnut skin extract. Results are from a single experiment performed in triplicate. **B** – Transmission-electron microscopy of thin sections of adult worms exposed to only medium (‘control’) or 1 mg/mL of hazelnut skin extract (‘CT’) for 24 hours. ‘cu’ – cuticle; ‘hy’ – hypodermis. Note the rough cuticle in worms exposed to CT (black arrows), and rupture and detachment of the hypodermis (white arrows). Scale bar =2 μM.

We also assessed the ability of hazelnut extract to reduce the motility of adult worms recovered directly from pigs – only a single extract was used here due to limited numbers of adult parasites. There was a clear dose-dependent reduction in motility, with worms exposed to 1 mg/mL of extract displaying little or no movement after 12 hours of incubation (Figure 5), and after 48 hours worms exposed to concentrations of ≥250 μg/mL showed clear signs of paralysis and structural damage including blebbing and a ‘rough’ cuticle. To explore further the structural damage caused by exposure to CT, the cuticle of worms exposed to the highest concentration of CT (1 mg/mL) was examined by transmission electron microscopy, which revealed clear damage with marked irregularity of the cuticular surface, in contrast to the smooth surface observed in control worms (Figure 5). In addition, the underlying hypodermis appeared to be torn and detached from the basal layer of the cuticle, with rupturing and lesions also observed within the hypodermis itself (Figure 5).

## Discussion

Continued reliance on mass drug administration with a limited number of synthetic anthelmintics has the potential to place heavy selection pressure on drug-resistant parasites, and widespread anthelmintic drug resistance is already a serious problem in many livestock production systems. The use of natural dietary compounds has the potential to be a complementary control option which may reduce this reliance on drug treatment, and slow the development of resistance. Here we have carried out a comprehensive *in vitro* assessment of the effects of CT from five different plant sources on one of the most prevalent parasites of pigs, *O. dentatum*.

We found that the development of free-living larvae was potently inhibited by all five extracts from CT-containing plants. This larval development assay is commonly used to screen potential anthelmintic compounds [26], however it has the drawback of focusing on stages of the parasite life cycle which are not exposed to the compound *in vivo*. Therefore, it cannot be automatically assumed that results observed with these free-living stages can be extrapolated to the parasitic stages found within the GI tract. Indeed, we found that a concentration (1 mg/mL) that completely inhibited larval development with all five extracts had only modest inhibitory effects against migration of exsheathed L3 parasites. Hazelnut skin, blackcurrant leaf and white clover flower extracts had no significant effects on larval migration, whilst extracts from willow bark and *Tilia* flowers did significantly inhibit migration but only by 20-30% - an effect considerably less than the 95% inhibition observed after incubation in ivermectin. Therefore, it is apparent that whilst some CT-containing extracts can inhibit migration of infective larvae, overall the anthelmintic effect is less pronounced against this early parasitic stage than against free-living larvae. Therefore, our results highlight the importance of assessing putative anthelmintic compounds against not only free-living parasites but also parasitic stages. The active compounds within *Tilia* flowers and willow bark were confirmed as CT by both PVPP-incubation and fractionation experiments, which demonstrated that anthelmintic activity was retained in CT fractions of 80-90% purity. It is interesting to contrast the results obtained here with our previous work with another pig nematode, *A. suum*, where incubation of L3 in comparable concentrations of CT led to a substantially higher inhibition of larval migration [17]. This suggests that not all helminth species are equally susceptible to the anthelmintic effects of CT, and careful assessment of activity is necessary before the suitability of these natural anti-parasitic compounds can be considered as viable control options in varying host-parasite systems.

Moulting of L3 to L4 parasites was significantly reduced after incubation in hazelnut skin, willow bark or *Tilia* flower extracts. Moreover, it was apparent that the motility of L4 was also reduced after incubation in extracts and fractions from the five plant sources. In the L3/L4 moulting assay, the motility and survival of the larvae was not affected by CT (data not shown), indicating that the reduction of development to L4 may be related to inhibition of specific metabolic processes involved in the moulting process [27]. To our knowledge, this is the first report that plant extracts can specifically inhibit the moulting process of a parasitic nematode, and further experiments are warranted to determine the mechanisms behind this inhibitory effect. It is also notable that adult worms appeared to be quite susceptible to the anthelmintic properties of CT. Further experiments with adult worms were not possible due to lack of parasite material, however the clear and rapid reductions in motility in adults exposed to the hazelnut skin extract indicate a potent effect. This suggests that dietary CT could potentially be used as a therapeutic option to specifically target adult worms and potentially prevent egg excretion and environmental contamination; however, further experiments will be necessary to explore this possibility. Overall, these experiments highlighted that while all stages of the parasite life cycle appeared to have some susceptibility to the plant extracts, clear differences between stages were observed. Interestingly, a recent study has also reported that a selection of medicinal plant extracts have increased activity towards *Caenorhabditis elegans* adults as compared to L3 or L4 stages, perhaps consistent with our results [28]. This has clear implications for the potential use of these plants for parasite control measures, as well as raising mechanistic questions about the observed anthelmintic activities.

Besides the quantity of CT, the structural characteristics of the polymers such as molecular weight and the ratio of different flavanol monomer units are known to affect anthelmintic activity [29,30]. In the current experiments, anthelmintic activity appeared to be associated with the higher molecular weight F2 fractions, suggesting that the anthelmintic activity in the extracts derives from CT with a high degree of polymerisation, consistent with previous studies with *A. suum* and *O. ostertagi* [17,29]. Moreover, extracts from *Tilia* flowers and willow bark were more potent in the L3 migration assays than hazelnut skin, white clover flower and blackcurrant extracts. Furthermore, *Tilia* flower, willow bark and hazelnut skin extracts significantly inhibited larval moulting, whereas white clover flower and blackcurrant extracts had no effect. Extracts and fractions derived from *Tilia* flowers also appeared to be most potent in the L4 motility assays, even when purified fractions were used with normalised concentrations of CT. Therefore, the observed differences in potency between the different acetone/water extracts do not appear to be related to the quantity of CT. Tannins in *Tilia* flowers, hazelnut skin and willow bark are comprised mainly of PC, whilst white clover flowers and blackcurrant leaves contain PD [17,31,32]. This suggests that PC-rich extracts/fractions may have higher activity towards *O. dentatum,* which is somewhat surprising given that PD are generally considered to have higher biological activity than PC (and the same applies to their corresponding, monomeric flavanol constituents) due to an extra hydroxyl group in the B-ring favouring increased hydrogen bonding with proteins [33]. Indeed, the monomeric constituents of PD (gallocatechin and epigallocatechin) have been shown to have higher anthelmintic activity than catechin and epicatechin against the ruminant nematodes *Trichostrongylus colubriformis and Haemonchus contortus* [30,34], and a correlation has also been noted between the proportion of PD in polymeric CT and anthelmintic activity against *O. ostertagi* [29]. Further studies with larger panels of well-characterised CT fractions will be necessary to determine if the PC:PD ratio plays a role in the anthelmintic effects observed here.

Given the overall lower efficacy of the isolated molecules against *O. dentatum* than comparable studies with *A. suum* [17], it may be that the mechanism of action of CT against *O. dentatum* is subtly different than to other helminths. At present, the anthelmintic mode-of-action of CT is not known, but is proposed to involve biochemical interactions between CT and proline-rich proteins on the nematode sheath or cuticle that interfere with both worm motility and feeding, and also key metabolic processes such as exsheathment (and perhaps also moulting, as suggested by our current data). This is supported by electron microscopy studies of worms exposed to CT that demonstrate direct structural damage to the cuticle [17,35], consistent with the ultrastructural changes we observed in the present study with adult *O. dentatum*. Such a mechanism would appear to be fairly non-specific and broad-spectrum in nature, hence it is interesting to note the differences in susceptibility between different nematodes. *O. dentatum* falls within the Strongyloididea superfamily of nematodes, a distinct family from the Ascaraidoidea (e.g. *A. suum*) and Trichostrongylidae (e.g. *T. colubriformis* and *O. ostertagi*) superfamilies [36], and this divergence may represent biological differences that determine susceptibility to CT. Comparative studies of these two nematodes, including transcriptomic and proteomic analyses of worms exposed to equivalent amounts of CT, may shed some light on the more precise mechanisms of the anthelmintic effect, and such studies are on-going in our laboratory. The marked differences in the response of *O. dentatum* during free-living development and adults to CT, compared to L3 stages, is also deserving of further studies to determine the mechanisms responsible.

An important consideration is how these *in vitro* results may be translated to *in vivo* studies in pigs. Experimental use of CT as an alternative anthelmintic in livestock has been characterised by two (somewhat overlapping) approaches, these being either short term consumption with the aim of either reducing establishment of incoming larvae or targeted therapeutic administration to remove adult worms [37,38], or long-term incorporation into the diet with the aim of disturbing key processes throughout the parasite life cycle, resulting in cumulative anthelmintic effects [39,40]. The fact that multiple processes in the life cycle of *O. dentatum* are affected by CT raises the possibility of a cumulative anthelmintic effect *in vivo*, whereby if a diet is consumed which contains CT, the additive sub-lethal effects against the different life cycle stages may reduce the viability and perhaps fecundity of worms as they mature within the host. In addition, the apparent susceptibility of adult worms to relatively low concentrations of CT raises the possibility of short-term feeding of CT as a complementary or alternative option to therapeutic drug treatment. Further *in vivo* studies to explore these options are necessary.

Moreover, the location of *O. dentatum* within the GI tract needs to be considered. Whilst there are a plethora of *in vivo* studies on the effects of CT-containing plants on worm infections in sheep and goats, as well as several rodent studies, most of these have focused on worms that reside in the stomach or small intestine, and thus there is only limited information on the effects of dietary CT upon large intestinal parasites. There is some evidence that pigs fed tannin-containing acorns (albeit hydrolysable tannins, quite different in structure to the CT tested here) have marked reductions in *O. dentatum* egg excretion [41]. However, sheep grazing the PC-rich forage *Lotus corniculatus* did not have reductions in burdens of *Oesophagostomum* spp., despite significant reductions in numbers of the abomasal parasite *H. contortus* and the small intestinal worm *Cooperia curticei* [42]. These authors speculated that changes to the structure of CT molecules in distal parts of the GI tract could affect anthelmintic activity. Whilst polymeric CT molecules are poorly absorbed from the GI tract [43], some fermentation and/or bacterial degradation of CT can occur in the monogastric large intestine [44-46]. This implies that dietary CT that are efficacious against parasites residing in the upper regions of the GI tract may not have comparable activity against parasites in distal regions, due to increased breakdown and a decrease in polymerization which may reduce potency. Therefore, further studies should focus on elucidating concentrations of CT in the local gut environments and whether polymeric CT retain their structure and anthelmintic activity through the entirety of the GI system.

## Conclusion

We have for the first time shown anthelmintic effects of CT-containing plant extracts and purified CT fractions against *O. dentatum*, and demonstrated that free-living/non-infective stages and adults appear to be highly susceptible to the effects of CT, whereas L4 are less susceptible and L3 are only modestly affected. Moreover, the moulting of L3 to L4 can be inhibited by CT, suggesting that specific, key processes in the parasite life cycle can be disrupted by CT. These data encourage further investigations to determine *in vivo* efficacy in pigs. In addition, further mechanistic studies, such as the relationship between the fine structure of CT molecules and anthelmintic activity, are also a high priority.

## Abbreviations

CT: Condensed tannins
PC: Procyanidins
PD: Prodelphinidins
